# The Relationship Between Achalasia and Esophageal Cancer: The Experience of a Tertiary Center

**DOI:** 10.5152/eurasianjmed.2022.21127

**Published:** 2022-02-01

**Authors:** Ömer Öztürk, Mustafa Kaplan, İlyas Tenlik, Volkan Gökbulut, Derya Arı, Yasemin Özin

**Affiliations:** 1 Gastroenterology Clinic, Ankara Bilkent City Hospital, Ankara, Turkey; 2 Gastroenterology Clinic, Kayseri Memorial Hospital, Kayseri, Turkey

**Keywords:** Achalasia, esophageal cancer, esophageal motility disorders

## Abstract

**Objective:** We examined the prevalence of esophageal cancer in 828 patients diagnosed with achalasia and the characteristics of patients who developed esophageal cancer.

**Material and Methods:** The demographic characteristics and medical records of the patients who were followed up with a diagnosis of achalasia between 1995 and 2019 were investigated from the patient files.

**Results:** The mean age of the patients was 51 ± 17.3, 390 of them were males (47.1%) and 438 were females (52.9%). The mean diagnosis age of the patients was 45.4 years. The median follow-up duration of the patients was 73 months (12-480). Esophageal cancer developed in 5 patients (0.6%) during follow-up. Three of these 5 patients had squamous cell carcinoma (60%) and 2 had adenocarcinoma (40%). Three of these patients were males (60%) and 2 were females (40%). The mean age of the patients was 68 (56-78), and cancer developed after a median of 156 months (24-216) after the achalasia diagnosis. Balloon dilation therapy was performed for the treatment of achalasia in all 5 patients who developed esophageal cancer.

**Conclusion:** Achalasia patients have an increased risk of developing esophageal cancer compared to the general population, and patients should be followed closely for cancer development.

## Main Points

- There is an increased risk of esophagus cancer development in achalasia patients compared to the general population.
- Esophageal cancer risk is higher in patients with achalasia over the age of 60 years.
- Esophageal cancer risk is higher in patients with achalasia who have been followed for more than 10 years.

## Introduction

Achalasia, the most commonly observed primary motor disorder in the esophagus, is characterized by relaxation disturbance in the lower sphincter of the esophagus and loss of peristalsis in the esophagus body. Although it is usually seen in the third and sixth decades, it can be observed at any age, and its incidence is 0.4-1.1 per 100 000 people.^1,2^ Achalasia, whose etiology is unknown and is usually idiopathic, has no definitive treatment. Today, its treatment is palliative. In treatments, the aim is to relieve the patients’ symptoms and to facilitate the transfer of food from the esophagus to the stomach by lowering the lower esophagus sphincter (LES) pressure.^3,4^

Esophageal cancer is one of the long-term complications of achalasia. Multiple risk factors are held responsible for the pathogenesis. In the pathogenesis of squamous cell esophagus carcinoma developing on the basis of achalasia, food retention in the lumen due to impaired motility in the esophagus body, impaired esophagus clearance, and relaxation in the lower esophagus are blamed. Food remains cause bacterial fermentation and stasis esophagitis. In a chronic period, this situation turns into dysplasia in the epithelium and then into cancer.^5-8^ In the pathogenesis of esophagus adenocarcinoma, the treatments performed for achalasia (such as balloon dilatation, Heller myotomy, per-oral endoscopic myotomy (POEM), and botulinum injection) lowering the LES pressure and resulting in increased gastroesophageal reflux diseases (GERD), esophagitis, and Barret esophagitis, are blamed.^9-11^ Despite the mechanisms mentioned above, the relationship between achalasia and esophageal cancer has not been clearly defined and is still a topic of discussion today. In a study, 3.3% of achalasia patients developed cancer,^12^ while this number was 0.75% in another study.^9^

We aimed to determine the esophagus cancer prevalence in the 828 patients followed up for achalasia in the present study.

## Materials and Methods

After our study protocol was examined and approved by Ankara Bilkent City Hospital’s ethics committee (No. E2-20-27), patient data were collected. Patients monitored between the years 1995 and 2019 in our gastroenterology clinic motility laboratory due to achalasia were retrospectively examined. The demographic characteristics, medical history, and records of the patients were reviewed from the files in our motility laboratory and hospital digital records. The patients’ achalasia diagnoses were confirmed with characteristic conventional manometry patterns (hypertensive LES pressures, complete and/or incomplete LES relaxation, aperistaltic esophagus body contractions) and barium swallow.^13,14^ At the initial diagnosis, esophagogastroduodenoscopy was performed to exclude organic pathologies and pseudo-achalasia in all patients.

In addition, the treatments performed on the patients due to achalasia (balloon dilatation, surgical, and medical treatments) were examined. All dilatations were performed with Rigiflex balloon dilators (30, 35, and 40 mm) (Microvasive; Boston Scientific, Watertown, Mass, USA).^15^ In patients monitored with balloon dilatation, repeated balloon dilatations between 1 and 5 times were performed in their follow-ups according to the patients’ clinical characteristics, Eckardt score, and manometry results.

Baseline symptoms of the patients were calculated with the Eckardt score (based on dysphagia, regurgitation, chest pain, and weight loss), and as classified in the previous study,^16^ those with an Eckardt score between 0 and 3 were considered as symptoms free, those with a score between 4 and 6 were considered as partial response, and those with a score of ≥6 were considered as symptoms continued. Follow-ups and treatment responses were also evaluated based on the Eckardt score.

Endoscopy was performed every 2-3 years (earlier if necessary) for patients with less than 10 years of illness, and an annual endoscopy was performed for those with more than 10 years of illness. Biopsies were taken from suspicious lesions. Patients whose cancer diagnosis was confirmed by biopsy were referred to the surgery and oncology departments. Patients who were under the age of 18, patients who had been monitored for less than 12 months, patients with a pseudo-achalasia^17^ diagnosis (lung adenocarcinoma, stomach cardia tumors, patients who developed a cardia relaxation disorder after the Nissen Fundoplication, and disorders that cause obstruction in the lumen due to external pressure to the esophagus) were excluded from the study.

Statistical Package for the Social Sciences 20.0 (IBM SPSS Corp.; Armonk, NY, USA) was used to run statistical analyses. Among the continuous numerical variables between the patients, those showing normal distributions were shown as mean ± standard deviation (SD), and those that were not distributed normally were shown as median, minimum-maximum values.

## Results

The results of a total of 828 achalasia patients were analyzed retrospectively. The demographic information and characteristics of the patients are presented in Table 1. The mean age of the patients was 51 ± 17.3, 390 (47.1%) of them were males and 438 (52.9%) were females. The mean diagnosis age of the patients was 45.4. The median follow-up duration of the patients was 73 (12-480) months. Endoscopic balloon dilatation was performed on 776 (94%) patients as first-line treatment. Balloon dilatation was applied to the patients a total of 1234 times. A total of 102 patients underwent surgical laparoscopic Heller myotomy + Dor fundoplication, 52 patients (6%) as the first-line treatment and 50 patients (6%) due to unresponsiveness to the balloon dilatation. Endoscopic balloon dilation was performed for 1 time on 502 patients (65%), for 2 times on 150 patients (19%), for 3 times on 77 patients (10%), for 4 times on 30 patients (4%), and for 5 times on 16 patients (2%).

While recurrent balloon dilation was successful in 93% of the patients, it failed in 7% of the patients. Remission was achieved in 79.4% of the patients with the first dilation. The second dilatation was performed at a median of 4 months after the first dilatation (1-140), and the third dilatation was performed at a median of 5 months after the second dilatation (1-60). Balloon dilatation was performed once in 2 of 5 patients who developed cancer, twice in 1 patient (114 months after the first dilatation), 3 times in 1 patient (1 month after the first dilatation and 37 months after the second dilatation), and 5 times in 1 patient (0-, 14-, 26-, 30-, and 23-month intervals).

Four patients underwent surgery due to the development of perforation after dilation. All perforations occurred after the first dilation of the patients. Three patients were dilated with a 30-mm balloon and 1 patient was dilated with a 35-mm balloon. Three of the four patients who developed perforation were over 50 years old, and 1 patient was 43 years old. Perforation did not occur in any patient who developed cancer.

Esophageal diameter was normal in 519 of 828 patients with achalasia on imaging at the time of diagnosis, diameter was increased in 302 patients, and sigmoid esophagus developed in 7 patients. Esophageal diameter was normal in 4 of 5 patients who developed cancer, and diameter was only increased in 1 of them.

Initial Eckardt score of 828 patients was 7.06 ± 2.15, lower esophageal sphincter pressure (LESP) was 37 ± 17.45. After treatments, Eckardt score was 3 in 420 patients, 4-6 in 323 patients, and >6 in 85 patients, while LESP was 13.13 ± 8.28. In 5 patients who developed cancer, the initial Eckardt score was 8.2 ± 2.28 and LESP was 40.4 ± 16.13. After treatment, Eckardt score was 3 in 2 patients, 4-6 in 2 patients, >6 in 1 patient, and LESP was 21.66 ± 11.54 (Table 2).

The data of patients who developed esophageal cancer are given in Table 3. Five patients developed esophageal cancer during follow-ups (0.6%). Three of these 5 patients had squamous cell carcinoma (60%), 2 had adenocarcinoma (40%). Three of these patients were male (60%), 2 were female (40%). The median age of the patients was 68 (56-78), and balloon dilation therapy was performed at a median of 2 (1-5) times in these patients who developed cancer. Cancer developed at a median of 156 months (24-216) after achalasia diagnosis. Those who developed adenocarcinoma had higher Eckardt scores at diagnosis, and Eckardt scores were higher after initial treatment. Cancer developed 5 years after diagnosis in all patients except 1. At the beginning endoscopy of the patient who developed cancer 24 months after the diagnosis, the esophagus was dilated and filled with food residues. The mucosa was normal in the endoscopy performed after the patient’s esophageal lumen was completely cleared. Esophageal wall thickness was normal in the thorax tomography taken for the advanced age of the patient and the exclusion of pseudo-achalasia. After balloon dilation therapy, the patient’s Eckardt score was <3. In the follow-up, the patient was diagnosed with esophageal cancer as the dysphagia complaints reappeared. When this patient was diagnosed with esophageal cancer, there was no local invasion and metastasis.

## Discussion

In this study, the rate of esophagus cancer development in achalasia patients was found as 6 in a 1000. Since this rate is higher than the general population, the necessity of closely monitoring achalasia patients was demonstrated in this study.

The incidence of esophagus cancers varies according to countries, even different geographical regions in the same country.^18^ While this rate is high in Asian countries, it is generally low in western and central Africa. Although esophageal cancer is the eighth most common cancer in the world and the sixth most common cause of death,^18,19^ it is not among the ten most frequently detected cancers in Turkey according to cancer statistics.^20^ Once again, the cancer types observed vary according to the regions as well. Adenocarcinoma rates are higher in Europe, while squamous cell cancer (SCC) rates are higher in Asia.^7,9^

The esophageal cancer frequency in Turkey has been determined with regional epidemiological studies. The esophageal cancer incidence was determined as 6 per 100 000 in women and 4 per 100 000 in men.^21^ While the median rate in this study was 5 per 100 000, in our study conducted with achalasia patients, it was observed that esophageal cancer developed at a rate of 6 per 1000, almost 100 times this rate.

In a study conducted with 117 139 cancer cases recorded for 25 years in the cancer database of Ege University Cancer Control Application and Research Center, 56% of the esophageal cancer cases were male, 44% were female, and the mean age was reported to be 60.^22^ In our study, the median age of our 5 patients who developed esophageal cancer was found as 68 and the male gender was more dominant, in concordance with the study above and many studies in the literature.

Since LES function decreases after balloon treatment and surgical treatment in achalasia patients, the rate of GERD increases. The most important risk factor for the development of adenocarcinoma is GERD.^9,11^ Similarly, the risk of cancer development will increase as the exposure to reflux increases after repeated balloon dilations, surgical interventions, and especially POEM for treatment.^23^ In our study, while balloon dilatation was performed 3 times in one and 5 times in the other during the treatment of the 2 patients who developed adenocarcinoma, balloon dilatation was performed once in 2 patients with SCC and 2 times in 1 patient. Patients who developed adenocarcinoma had high Eckardt scores at diagnosis and after the initial therapy, and therefore they need repeated balloon dilations during their follow-up. The risk of acid exposure of the esophagus increases with repeated balloon dilations (23). Patients with adenocarcinoma had more PD than those with SCC. Therefore, exposure of esophagus to more acid may increase the risk of adenocarcinoma. This situation could not be clearly evaluated since our study was retrospective and 24-hour pH metrics were not performed after treatments and during follow-up.

Surgery was not performed in the treatment of any of our patients who developed cancer. In a study conducted by Zendehdel et al^9^ with 2896 achalasia patients in Sweden, 22 (0.75%) patients developed esophageal cancer (15 SCC and 7 adenocarcinoma) and only 1 patient who developed adenocarcinoma underwent surgical treatment for achalasia. Additionally, Markar et al^23^ reported that balloon dilatation as primary therapy was associated with increased cancer risk. On the other hand, Zaninotto et al^24^ reported that SCC is developed in 1.8% of achalasia patients who had underwent laparoscopic Heller-Dor (LHD) myotomy, with a median follow-up of 18.3 years. However, the same researchers recently reported 2 cases of SCC out of 1001 achalasia patients (approximately 0.2%) who had underwent LHD myotomy, with a far shorter follow-up.^25^ There is hardly evidence, therefore, that PD carries a higher risk of cancer compared to myotomy. It may be only a matter of time. Possibly, only further follow-up of the patients of the European Achalasia Randomized Trial^26^ and other^27^ studies may provide an answer to that.

Whether to perform surveillance for esophagus cancer in achalasia patients is also one of the current discussions. Most studies have shown that esophageal cancer in patients with achalasia is usually seen after 10 years.^9,12,28,29^ However, the American Gastrointestinal Endoscopy Society (ASGE) and the American Gastroenterology Association (ACG) do not recommend surveillance in achalasia patients.^19,30^ In a cohort study where data of 11 years were analyzed retrospectively in the British society,^23^ 7487 patients with achalasia were evaluated. Esophageal cancer developed in 101 (1.3%) patients. The median cancer development duration was 3 (1-11) years, and 71.3% of the patients who developed cancer were over the age of 60. There was no difference between cancer development and gender. In a meta-analysis conducted by Tustumi et al^31^ in 2017, the prevalence of esophagus cancer was reported as 28 in 1000 achalasia patients. If surveillance is to be performed in patients with achalasia, it is recommended to start 10 years after the onset of the achalasia symptoms in the stated study. In our study, 80% of the patients who developed cancer were over the age of 60 and developed cancer at a median of 156 months after the diagnosis. In the patient who was diagnosed with cancer 24 months after the diagnosis of achalasia, the diagnosis of achalasia was probably delayed due to the dilated esophagus at the time of presentation. In a study conducted by Leeuwenburgh et al^12^ in the Netherlands, 15 of 448 achalasia patients developed esophageal cancer (12 squamous cell cancer and 3 adenocarcinoma), and 10 of the patients who developed cancer were male and 5 were female. The median age of patients with cancer was 71 years, and cancer development was observed after 13 (1-36) years. Although ASGE and the ACG do not recommend surveillance in achalasia patients,^19,30^ esophageal cancer risk appears to be higher in patients over the age of 60 and who have been followed for more than 10 years.

The limitations of this study are that it is retrospective and we do not have information about their surveys since patients are not followed up by us after the cancer diagnosis. In addition, since the study was retrospective, other risk factors for esophageal cancer, such as smoking, dietary habits, alcohol use, and obesity, could not be questioned. Also, achalasia diagnoses were determined by conventional manometry, and achalasia subtypes could not be detected in patients who developed cancer because high-resolution manometry could not be used. Due to the retrospective design of the study, a routine pH meter could not be applied to patients in this study. Unfortunately, the results of the patients who had a pH meter were not reached and the information on whether these patients regularly took proton pump inhibitors or other anti-acid drugs could not be reached.

In conclusion, there is an increased risk of esophagus cancer development in achalasia patients compared to the general population. We do not suggest routine cancer surveillance, but if surveillance is to be done, we suggest that it is done with patients who are over the age of 60 and have been followed for more than 10 years.

## Figures and Tables

**Table 1.** Demographic and Characteristics of the Patients

Age, mean	51 ± 17.3
Male/Female (%)	438 (52.9%)/390 (47.1%)
Diagnosis age of achalasia, mean	45.4 ± 15.4
Disease follow-up time due to achalasia, median, months (min-max)	73 (12-480)
**Treatment options for achalasia** Patients who underwent balloon dilatation in the first treatment Patients who underwent surgical myotomy in the first treatment Patients who underwent surgical myotomy after balloon treatment	776/828 52/828 50/766
**Patients who underwent balloon dilatation in the first treatment** The number of patients who received 1 dilatation The number of patients who received 2 dilatations The number of patients who received 3 dilatations The number of patients who received 4 dilatations The number of patients who received 5 dilatations	776 503 (65%) 150 (19%) 77 (10%) 30 (4%) 16 (2%)
**Esophageal cancer** Squamous cell cancer/adenocarcinoma Male/female Age, median (min-max) Follow-up time, median (min-max) Balloon dilation number, median, (min-max)	5/828 (0.6%) 3/5 (60%)/2/5 (40%) 3/5 (60%)/2/5 (40%) 68 (56-78) 156 (24-216) 2 (1-5)

**Table 2.** Imaging and Manometry Findings of Patients at Diagnosis

	All Patients (n = 828)	Patients with Cancer (n = 5)
Endoscopy finding at diagnosis (n = 828) Normal esophagus Dilated esophagus Sigmoid esophagus	519 302 7	4 1 -
Eckardt score, mean	7.06 ± 2.15	8.2 ± 2.28
Eckardt score after therapy Eckardt score ≤ 3 Eckardt score 4-6 Eckardt score > 6	420 323 85	2 2 1
Lower esophageal sphincter pressure (mmHg), mean At diagnosis After therapy	37 ± 17.4513.13 ± 8.28	40.4 ± 16.1321.66 ± 11.54

**Table 3.** Patients with Esophageal Cancer

	Age (years)	Gender	Cancer Type	Follow-Up Period due to Achalasia (Months)	Treatments for Achalasia	Eckardt Score at Diagnosis and After Initial Therapy	
Patient 1	78	Male	Squamous cell cancer	216	Twice balloon dilation	7	4-6
Patient 2	65	Female	Squamous cell cancer	180	Once balloon dilation	5	<3
Patient 3	77	Male	Squamous cell cancer	24	Once balloon dilation	9	<3
Patient 4	64	Female	Adenocarcinoma	156	Five times balloon dilation	9	4-6
Patient 5	56	Male	Adenocarcinoma	62	Three times balloon dilation	11	>6
